# Expression and Prognostic Analysis of Integrins in Gastric Cancer

**DOI:** 10.1155/2020/8862228

**Published:** 2020-11-30

**Authors:** Jun-Fu Wang, Ye Wang, Si-Wen Zhang, Ye-Yang Chen, Yue Qiu, Shao-Yi Duan, Bo-Pei Li, Jun-Qiang Chen

**Affiliations:** Department of Gastrointestinal Surgery, The First Affiliated Hospital of Guangxi Medical University, 6 Shuangyong Road, Nanning 530021, Guangxi Zhuang Autonomous Region, China

## Abstract

**Background:**

Integrins are involved in the biological process of a variety of cancers, but their importance in the diagnosis and prognosis of gastric cancer (GC) is still unclear. Therefore, this study aimed at exploring the significance of ITG gene expression in GC to evaluate its diagnosis and prognosis.

**Methods:**

GEPIA data were used to evaluate the mRNA expression of ITG genes in GC patients. The prognostic value of these genes was assessed by analyzing their mRNA expression using the Kaplan–Meier curve. The biological function of ITG genes was evaluated by GC tissue sequencing combined with GSEA bioinformatics. Based on the sequencing data, ITGA5 with the largest expression difference was selected for verification, and RT-PCR was used to verify its mRNA expression level in 40 pairs of GC and normal tissues.

**Results:**

ITG (A2, A3, A4, A5, A6, A11, AE, AL, AM, AV, AX, B1, B2, B4, B5, B6, and B8) was highly expressed in GC tissues, while ITGA8 was low, compared with their expression in normal tissues. RNA-seq data shows that ITG (A2, A5, A11, AV, and B1) expression was associated with poor prognosis and overall survival. In addition, combined with the results of GC tissue mRNA sequencing, it was further found that the differentially expressed genes in the ITGs genes. ITGA5 was highly expressed in GC tissues compared with its expression in normal tissues, as evaluated by qRT–PCR (*P* < 0.001) and ROC (*P* < 0.001, AUC (95% CI) = 0.747 (0.641–0.851)), and confirmed that ITGA5 expression was a potential diagnostic marker for GC. Bioinformatics analysis revealed that the signaling pathway involved in ITGA5 was mainly enriched in focal adhesion, ECM-receptor interaction, and PI3K-AKT and was mainly involved in biological processes such as cell adhesion, extracellular matrix, and cell migration.

**Conclusion:**

This study suggested that ITGs were associated with the diagnosis and prognosis of GC and discovered the prognostic value and biological role of ITGA5 in GC. Thus, ITGA5 might be used as a potential diagnostic marker for GC.

## 1. Introduction

Gastric cancer (GC) is one of the most common malignant tumors worldwide. Although the diagnosis of GC has made progress, most patients in China are in the middle and advanced stages at the time of diagnosis. The outcomes after surgery, radiotherapy, and chemotherapy are poor, and the five-year survival rate is less than 30% [1]. The current parameters to predict GC diagnosis and prognosis are not precise enough. Therefore, there is an urgent need to find potential new biomarkers and prognostic indicators to create individualized treatment in clinical practice.

Integrins (ITGs) regulate a variety of biological activities but also human diseases, including tumor progression [2]. ITGs are heterodimeric glycoproteins formed by two subunits, *α* and *β* consisting of 18 *α* subunits and 8 *β* subunits forming transmembrane surface receptors [3]. The ITG family proteins allow the attachment of the cells to the extracellular matrix, mediate the cell-to-cell adhesion, maintain the morphology of cells, and affect several processes including movement and phagocytosis [4]. Another function is to activate a bidirectional signal mediated by the release of stimulating factors from the cell. Indeed, ITGs transmit the signal from the intracellular area to the extracellular area by combining themselves with various cytoplasmic proteins to form a complex that mediate signal transduction, mainly including FAK-PI3K-AKT, FAK-STAT1, FAK-Ras-MAPK, and other pathways. This signal transduction may affect the expression of genes involved in cell proliferation, migration, invasion, differentiation, and apoptosis. Thus, they might be considered a new strategy in the diagnosis and treatment of tumors [5–7].

At present, some studies on the ITG genes in GC are available, and many scientists discovered that the polymorphism of the ITG (A1, A2, A3, A6, B3, B4, B4, and B5) gene is closely related to the risk of GC [8]. Chuang et al. demonstrate that the block of ITGA2 inhibits the migration and apoptosis of GC cells, thus concluding that it may be considered as a target in GC therapy [9]. The coordination of the mir-30a-ITGA2 axis is another important mechanism in the occurrence and development of GC precancerous lesions [10]. Cao et al. revealed that ITGA5 may be a potential biomarker and therapeutic target of GC through bioinformatics analysis [11]. Yang et al. found that ITGA6 may become a new biomarker for the prediction and treatment of GC, as evaluated by immune indicators and prognostic prediction model of GC [12]. The analysis of the GC genome methylation pattern revealed that genome methylation promotes the expression of ITGAM, suggesting that it might be a candidate gene in GC diagnosis [13]. ITGAV can promote the proliferation, invasion, and migration of GC cells [14].

Despite all these discoveries, the role of ITGs in GC is still unclear. Therefore, this study aimed to explore the expression of ITG genes in GC and the biological processes and signaling pathways involved by bioinformatics and high-throughput sequencing, to clarify the potential value of ITG genes in the diagnosis and prognosis of GC.

## 2. Materials and Methods

### 2.1. Patients and Sample Collection

All tissue samples were collected from 50 patients with gastric cancer treated in the Department of Gastrointestinal Gland Surgery of the First Affiliated Hospital of Guangxi Medical University from September 2018 to December 2019. All patients were diagnosed by pathology under electronic gastroscope before operation. Patients with preoperative radiotherapy and chemotherapy and other systematic tumors or tumor metastasis to the stomach were excluded. Postoperative pathology was confirmed by tumor pathologists. Tissue samples were quickly frozen at −80°C in vitro for follow-up experiments. All the specimens were matched with adjacent normal tissues, of which 10 pairs of samples were used for tissue mRNA-seq and 40 pairs for RT-qPCR verification. This study was approved by the Ethics Review Committee of the First Affiliated Hospital of Guangxi Medical University. The patients all signed the written informed consent form.

### 2.2. ITG Gene Expression Using GEPIA Database

The expression of the ITG genes was analyzed using the Gene Expression Profiling Interactive Analysis (GEPIA) database (http://gepia.cancer–pku.cn/). Normal samples and tumor samples were selected from the TCGA database, and log_2_ FC >0.5 and a *P*-value <0.05 were used as screening conditions. The expression of ITG genes in GC tissues at different development stages was explored. The expression values were converted to log_2_ (TPM + 1) to draw the box plot. According to the GC tissue RNA-seq data in the UCSC XENA database, the heat map of ITG gene expression was analyzed and drawn (http://ualcan.path.uab.edu/). The changes of ITGs were analyzed by the TCGA comprehensive analysis platform cBioPortal.

### 2.3. Prognostic Analysis of the ITG Genes Using the Kaplan–Meier Plotter

Data of the K-M Plotter database (http://kmplot.com/analysis) were derived from GEO, EGA, and TCGA. According to the mRNA-seq data of TCGA, the overall survival (OS) and relapse-free survival (RFS) according to the ITG gene expression were mapped out, and the clinical data of different tumor stages and grades were recorded for further analysis.

### 2.4. mRNA-seq of GC Tissue

GC tissue and adjacent normal tissues were collected from 10 GC patients undergoing routine surgery, and high-throughput sequencing was carried out at the Shanghai Biotechnology Co., Ltd., and an mRNA sequencing library was constructed using VAHTSTM mRNA-seq V2 Library Prep Kit for Illumina® (San Diego, California, USA). Finally, the sequencing was performed on the HiSeq XTen sequencer, and DESeq was used for the analysis of samples with biological duplicates. In order to obtain genes with significant difference, the screening conditions were set at a *q* value <0.05, the difference as |fold change| > 2, and the matched genes were considered as genes with significant differentiation. Bioinformatics methods were used to analyze the biological processes, and signaling pathways that differentially expressed genes may participate. The clusterProfiler was used for functional enrichment analysis.

### 2.5. ITGA5 mRNA Expression by qRT-PCR

TRIzol reagent was used to extract total RNA from tissues, and cDNA was obtained using a reverse transcription kit from Takara Biotechnology Co., Ltd. SYBR-Green I fluorescence detection kit and a 7500 Real-Time PCR System were used for qRT-PCR to detect ITGA5 mRNA expression. The reaction conditions were as follows: 95°C, 30 s; 95°C, 5 s; 63°C, 30 s; 40 cycles. The primers were synthesized by Sangon. The GAPDH primer sequence was as follows: F 5′-TGACTTCAACAGCGACACCCA-3′, R 5′-CACCCTGTTGCTGTAGCCAAA-3′, while the ITGA5 primer sequence was as follows: F 5′-GGCTTCAACTTAGACGCGGAG-3′, R 5′-TGGCTGGTATTAGCCTTGGGT-3′.

### 2.6. Gene Enrichment Analysis

In order to further explore the potential value of the ITGA5 gene in biological processes and pathways, the data were downloaded from TCGA, the GSEA desktop software application (http://www.broadinstitute.org/gsea/index.jsp) was used to perform the analysis, and the potential mechanisms were investigated by GSEA (http://software.Broadstitute.org/gSEA/index.jsp) through Molecular Signatures Database (MSigDB) c2 (c2.cp.kegg.v6.2.symbols.gmt) and c5(c5.all.v6.2.symbols.gmt). The enrichment analysis of GSEA was considered statistically significant when *P* < 0.05 and FDR < 0.25.

### 2.7. Statistical Analysis

Statistical analysis was performed using GraphPad 7.0 software, and the relative expression of ITGA5 mRNA in tissues was analyzed by paired *t*-test and one-way analysis of variance. *P* < 0.05 was considered statistically significant. ROC curve analysis is a common method to evaluate the performance of tumor diagnostic markers. Therefore, the ROC curve was used to detect the accuracy of the ITGA5 gene as a biomarker for GC prediction.

## 3. Results

### 3.1. mRNA Expression of Several ITGs in GC

**Figure 1:**
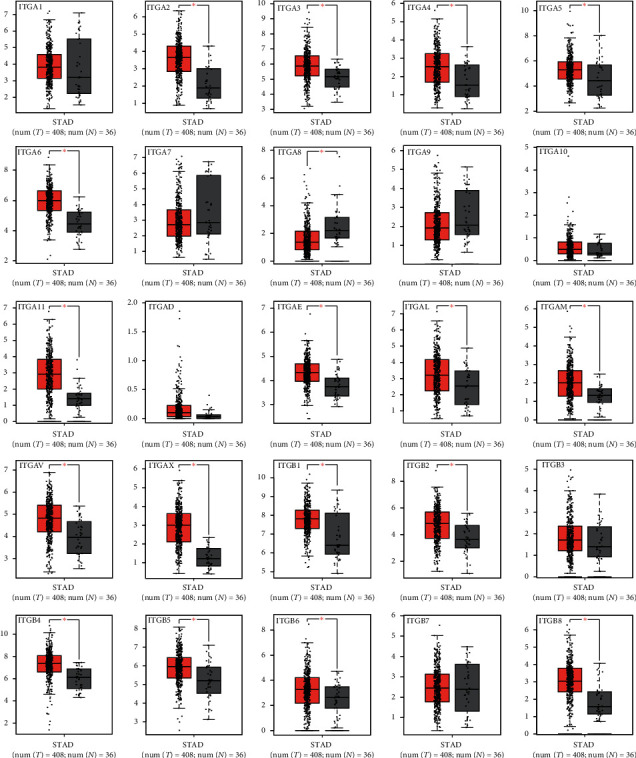
mRNA expression of ITGs in GC and normal tissues according to GEPIA; ^*∗*^*P* < 0.05.

**Figure 2:**
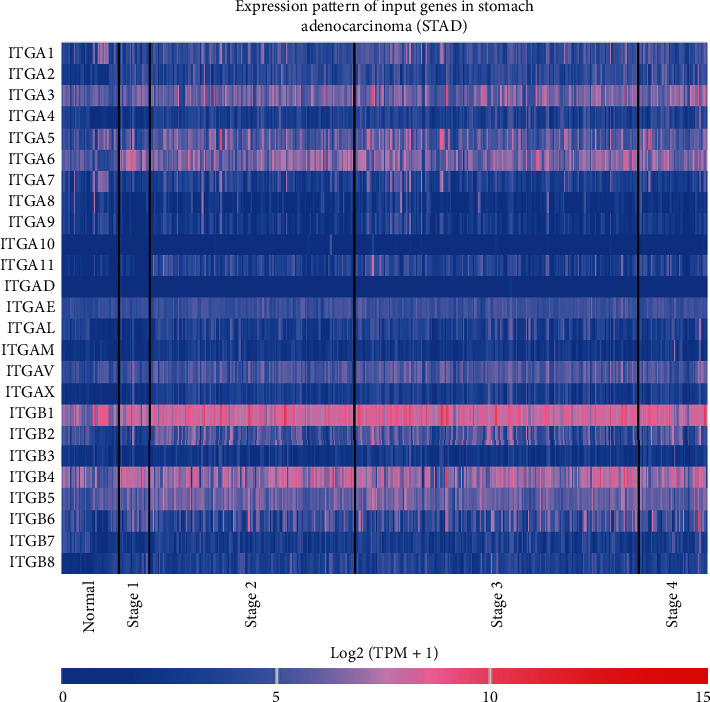
Expression of ITGs in GC patients according to the UCSC XENA database. The red color indicates high expression; the blue color indicates low expression.

**Figure 3:**
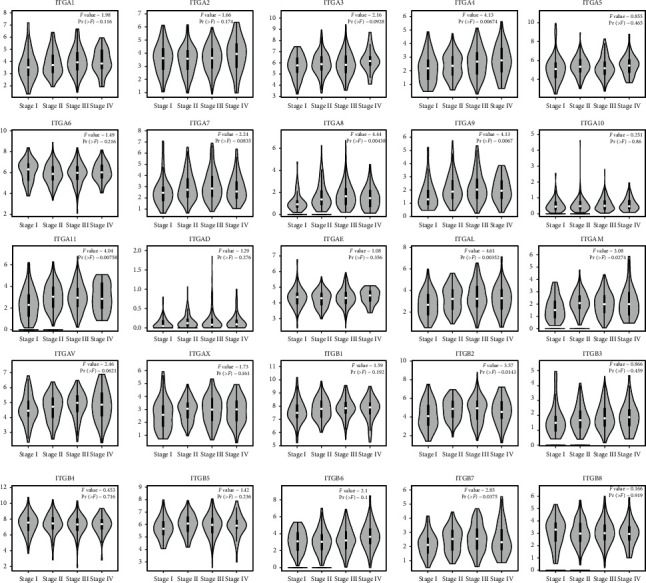
Expression of ITGs genes in different stages of GC according to GEPIA.

**Figure 4:**
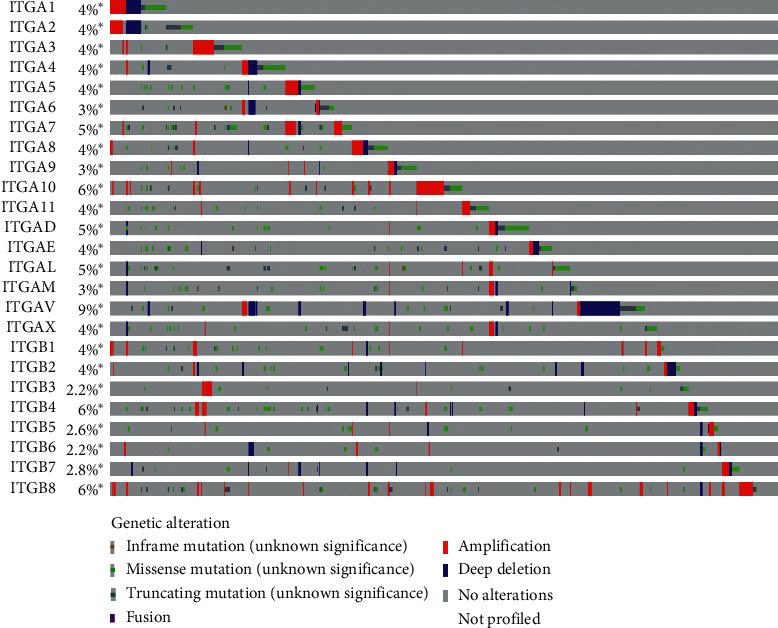
ITGs gene changes according to the TCGA database. ITGAV had the highest mutation rate (9%), and deep deletions and missense mutations were the main represented ones.

The mRNA expression of ITG (A2, A3, A4, A5, A6, A11, AE, AL, AM, AV, AX, B1, B2, B4, B5, B6, and B8) and other genes in 408 GC tissue samples and 36 normal gastric tissues were higher than those in the adjacent normal tissues (*P* < 0.05) (Figure 1). ITGA8 expression in GC tissues was significantly lower than that in normal tissues (*P* < 0.05). The expression of ITGs in cancer tissues and adjacent normal tissues is shown in Figure 2. The relationship between ITG genes and GC progression is shown in Figure 3, in which genes such as ITG (A4, A8, A9, A11, AL, B2, and B7) increased with the increase of tumor progression (*P* < 0.05). The analysis of ITG genes revealed that the mutation rate of ITGAV was as high as 9%, mainly due to a deep deletion and missense mutations. ITGB3 and ITGB6 genes had the lowest gene mutation rate, which was only 2.2% (Figure 4).

### 3.2. Prognostic Value of ITGs in GC Patients

**Figure 5:**
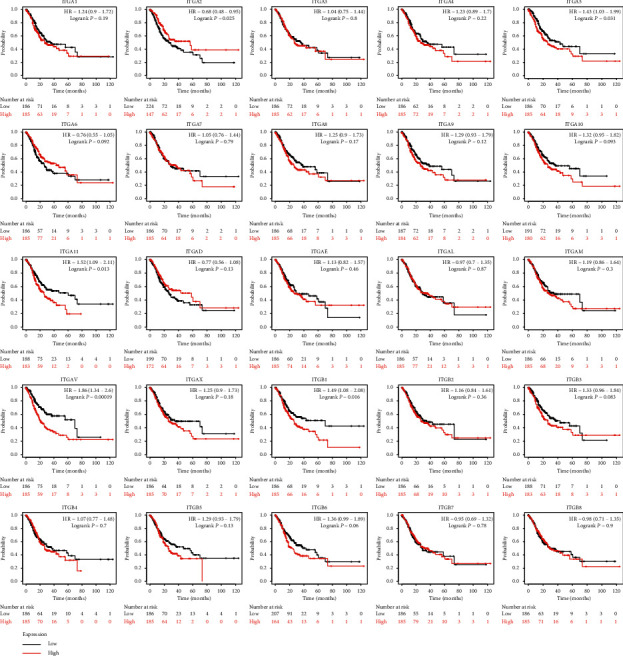
OS according to the expression of ITG genes based on RNA-seq data in Kaplan–Meier plotter.

**Figure 6:**
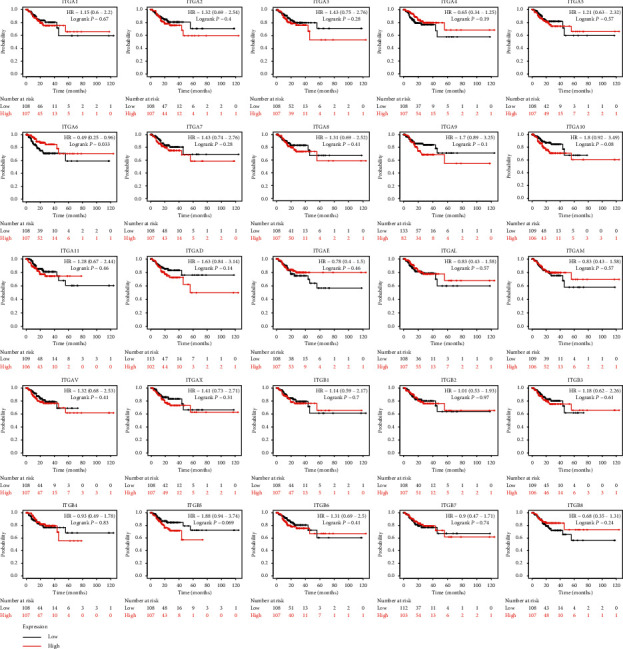
RFS according to the expression of ITG genes based on RNA-seq data in Kaplan–Meier plotter.

The RNA-seq data in the TCGA database revealed the prognostic value of the mRNA expression of the ITG genes in GC. ITGA2 (HR = 0.68, *P* < 0.025), ITGA5 (HR = 1.43, *P* < 0.031), ITGA11 (HR = 1.52, *P* < 0.013), ITGAV (HR = 1.86, *P* < 0.001), ITGB1 (HR = 1.49, *P* < 0.016) were higher in patients with short OS compared to their expression in patients with longer OS, and the OS of GC patients with high expression of these genes was significantly shortened (Figure 5). The expression of ITGA6 (HR = 0.49, *P* < 0.033) was related to GC recurrence. The RFS of patients with high expression of this gene was significantly longer than that of patients with low expression of this gene (Figure 6).

The subgroup analysis showed that ITGA6 was associated with poor OS in stage II. ITGA5 and ITGAV were associated with poor OS in stage III. ITGA5 and ITGAV were associated with poor OS in stage IV (Table 1). The prognosis associated with the expression of ITG genes was statistically analyzed in different cancer stages (Table 2). ITG (A2, A6, AE, and AV) genes were related to poor OS in stage II, and ITG (AE, AL, AV, B1, and B5) genes were related to poor OS in stage III.

### 3.3. ITGs with Differential Expression Detected by Sequencing of Tissue Samples

**Figure 7:**
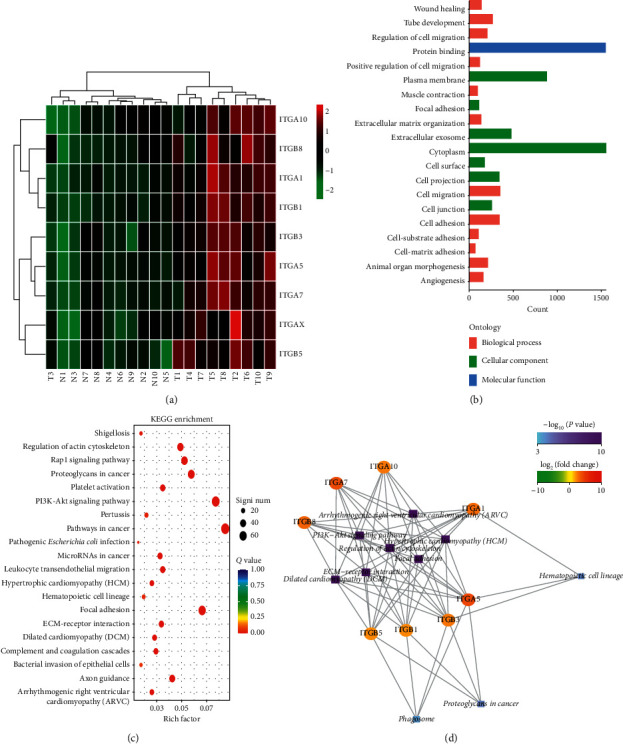
Differentially expressed ITG genes screened out according to GC tissue sequencing results. (a) Differentially expressed ITG genes in GC tissues shown in the heat map; (b) the biological processes involved in differentially expressed ITG genes; (c)-(d) the signaling pathways involved in differentially expresses ITG genes.

Using a *q* value <0.05 and the difference |fold change| > 2 as the standard to identify differentially expressed genes, our results showed that ITG (A1, A5, A7, A10, AX, B1, B3, B5, and B8) was significantly differentially expressed among ITGs, and the heat map showed that differential ITGs were significantly highly expressed in cancer tissues (Figure 7(a)). According to the GO annotation analysis of the different ITGs screened from the two groups (gastric cancer group and adjacent normal tissue group), the ITGs were significantly upregulated in functions such as cell adhesion, extracellular matrix, and cell migration (Figure 7(b)). KEGG annotation analysis showed that ITG signaling pathways were mainly enriched in focal adhesion, ECM-receptor interaction, PI3K-AKT, and cancer related pathways (Figures 7(c) and 7(d)).

### 3.4. ITGA5 mRNA Expression Verification in Clinical Samples

**Figure 8:**
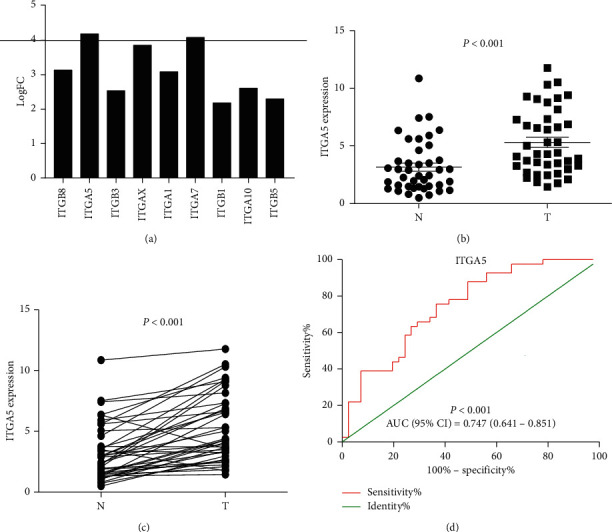
ITGA5 relative expression in normal tissues and cancer tissues detected by RT–PCR. (a) According to the standard of *q*-value <0.05 and difference multiple |fold change| > 2, the differentially expressed genes in ITGs were screened out from 10 pairs of gastric cancer tissue samples, and the fold change of ITGA5 was the highest; (b) and (c) ITGA5 mRNA relative expression in GC tissues (*n* = 40, *t*-test); (d) the diagnostic ROC curve of the distribution of ITGA5 relative expression.

Sequencing results showed that ITGA5 had the highest fold change difference (Figure 8(a)); thus, it was selected for clinical verification. In order to further study the expression of ITGA5 in GC and its diagnostic value, *q* RT-PCR was used to verify the expression of ITGA5 in tissues (40 GC tissues and 40 adjacent normal tissues). Our results showed that ITGA5 expression in GC tissues was significantly higher than that in the adjacent normal tissues (*P* < 0.001) (Figures 8(b) and 8(c)). The ROC curve was used to study the potential diagnostic role of ITGA5 in clinical samples, and the results showed that ITGA5 has a significant meaning (*P* < 0.001, AUC (95% CI) = 0.747 (0.641–0.851)) (Figure 8(d)).

### 3.5. ITGA5 Functional Enrichment Analysis

**Figure 9:**
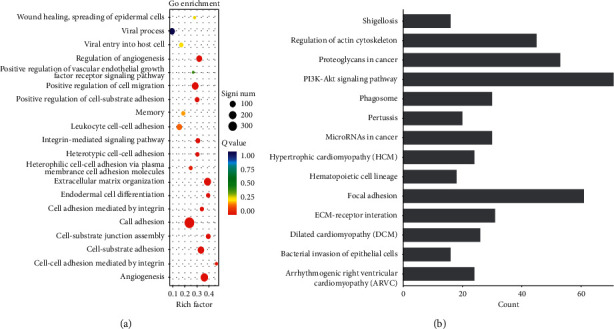
KEGG and GO enrichment analysis results of ITGA5 gene in GC tissue sequencing data, showing the biological processes and signaling pathways involved in ITGA5.

The signaling pathways involved in ITGA5 were mainly enriched in focal adhesion, ECM-receptor interaction, PI3K-AKT, and cancer-related pathways, mainly involved in protein connection, cell adhesion, focal adhesion, cell matrix receptor interaction, and other biological processes. Among them, focal adhesion, ECM-receptor interaction, and PI3K-AKT were the most significant; thus, our following research focused on these signaling pathways (Figure 9).

### 3.6. GSEA

**Figure 10:**
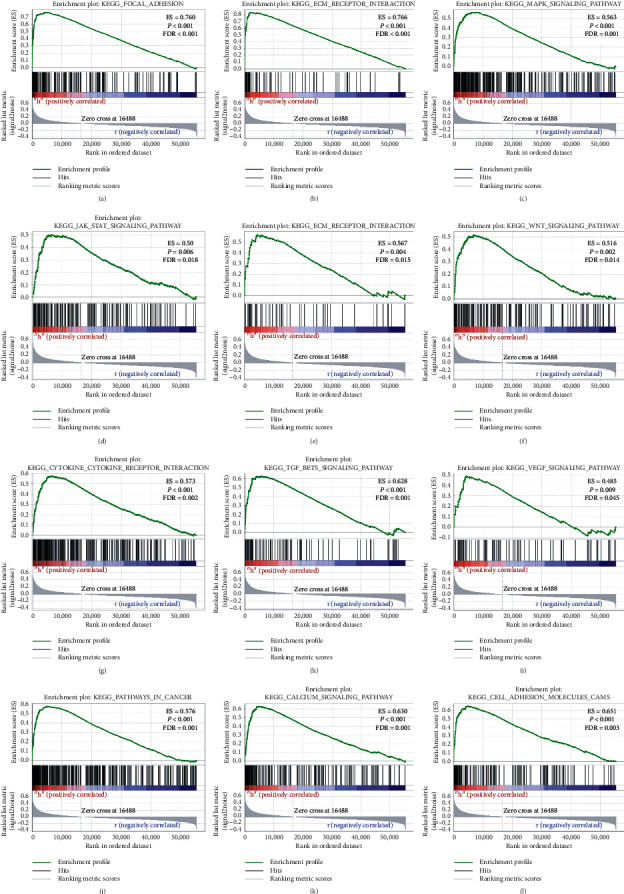
Gene enrichment analysis results of ITGA5 in GC patients according to the TCGA database. A–M, GSEA results of the C2 gene set of the ITGA5 high expression group.

**Figure 11:**
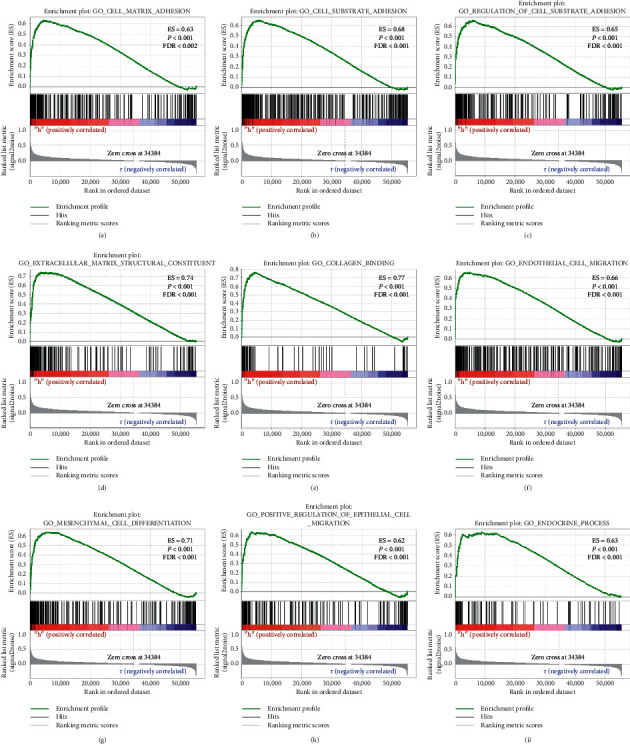
Gene enrichment analysis results of ITGA5 in GC patients according to the TCGA database. A–M, GSEA results of the C5 gene set of the ITGA5 high expression group.

Further verification based on GSEA showed that the C2 gene set indicated that the high expression of ITGA5 might be closely related to focal adhesion, ECM receptors, MAPK, JNK, MTOR, WNT, FGF-*β*, VEGF, and cancer pathways (Figure 10), and the C5 gene set might be mainly involved in extracellular matrix, cell migration, cell secretion, and other biological processes (Figure 11).

## 4. Discussion

In this study, GEPIA database and Kaplan–Meier plotter were used to explore the expression of ITG genes and their significance in the prognosis of GC, and most of them were highly expressed in GC. According to the RNA-seq data, the high expression of ITG (A2, A5, A11, AV, and B1) in the ITGs family was negatively correlated with the patient OS. The genetic mutations in ITGs revealed a common cause of genetic changes. The combination of the results of GC tissue sequencing and GSEA data analysis demonstrated that ITGs might participate in the biological processes and signaling pathways related to cancer. ITGA5 expression in GC was confirmed by qRT-PCR, and the confirmed results were consistent with the bioinformatics and tissue sequencing data. The AUC ROC curve of ITGA5 relative mRNA expression (95% CI) was 0.747 (0.641–0.851); *P* < 0.001. Thus, the diagnostic value of ITGA5 in GC was determined.

Our results on ITGs suggested that most ITGs were related to tumor diseases. Indeed, some previous studies reported the clinical outcome associated to ITGs in cancer patients, but these studies usually assess the prognostic value of individual ITGs on small sample sizes. Our research was based on the analysis of relatively large data samples of TCGA; therefore, more reliable results were achieved. In this study, previous studies and the role of ITGs genes in cancer were mainly discussed.

ITGA1 is highly expressed in pancreatic cancer [15], colorectal cancer [16, 17], and GC [18]. Another study found that it was significantly related to colon tumor metastasis and clinical staging and can promote the invasion, migration, and tumorigenicity of colon cancer cells. In vivo and in vitro experiments proved that ITGA1 is an oncogene and may be considered a new target for colon cancer diagnosis and treatment [16, 17]. ITGA1 gene is also involved in peritoneal metastasis of GC cells [19]. The assessment of GC risk in South Korea′s population revealed that the polymorphism and haplotype of ITGA1 gene were significantly associated with increased GC risk [8]. However, the results of this study showed that ITGA1 expression was not significant to evaluate the prognosis of GC patients.

ITGA2 is highly expressed in GC [9], liver cancer [20], and pancreatic cancer [21]. ITGA2 gene polymorphism in GC is associated with an increased risk of GC [8]. Chuang et al. found that blocking ITGA2 inhibits the migration and apoptosis of GC cells, suggesting that this gene may be the target of GC therapy [9]. The coordination of the mir-30a-ITGA2 axis may be an important mechanism in the occurrence and development of GC precancerous lesions and intestinal GC [10]. Our results in this study were consistent with these findings. According to this study, ITGA2 is highly expressed in GC and is negatively correlated with the OS of GC patients.

ITGA3 is highly expressed in bladder cancer [22], intrahepatic cholangiocarcinoma [23], pancreatic cancer [24], and nasopharyngeal carcinoma [25]. Its high expression in intrahepatic cholangiocarcinoma and nasopharyngeal carcinoma is negatively correlated with patient OS and can promote the proliferation of intrahepatic cholangiocarcinoma cells and cell cycle, thus, potentially representing a new target in the treatment of intrahepatic cholangiocarcinoma [23, 25]. Bioinformatics analysis revealed that the expression of ITGA3 can be used as a diagnostic and prognostic marker for pancreatic cancer [24], but its expression in GC is still unclear. According to our research, ITGA3 was highly expressed in GC, but no significant correlation was found between the expression of this gene in GC and patient prognosis.

ITGA4 is highly expressed in high-risk neuroblastoma, and it can mediate cell invasion and migration in high-risk neuroblastoma and melanoma. It is considered as a key factor in tumor cell invasion and migration and plays an important role in cell-cell and cell-extracellular matrix interaction [26]. In this study, ITGA4 was highly expressed in GC, and its expression was increased with the progression of GC staging, but it was not correlated with patient OS.

ITGA5 is highly expressed in oral squamous cell carcinoma [27], pancreatic cancer [28], GC [11], and colorectal cancer [29], and its high expression is negatively correlated with patient OS. Some studies revealed the prognostic role of ITGA5 in non-small cell lung cancer through bioinformatics, which resulted as an independent prognostic predictor, and the five-year survival rate of patients with high ITGA5 expression was significantly reduced [30]. The high expression of ITGA5 was significantly related to poor prognosis in patients with peritoneal metastasis of ovarian cancer, and the OS time of patients with advanced ovarian cancer and with high ITGA5 expression was relatively shorter [31]. The GC bioinformatics research revealed that the high ITGA5 expression reduced the OS rate in GC patients. Therefore, it may be considered a potential biomarker and therapeutic target to combat GC, and it may mainly play an important role in the FAK and ECM receptor signaling pathways [11]. This study found that ITGA5 was highly expressed in GC and was related to a poor prognosis of GC patients. Thus, the results in this study were consistent with the aforementioned previous findings but lack the experimental verification in GC.

ITGA6 gene polymorphism is associated with increased GC risk [8]. Yang et al. found that ITGA6 may become a new biomarker and treatment target for GC according to their established GC prognostic immune indicators and prognostic prediction model [12]. This study was consistent with their study, since also in our study ITGA6 was highly expressed in GC, but the survival curve obtained by the RNA-seq data analysis showed opposite results to the ones of previous studies. Thus, a further clinical verification is needed.

ITGA7 is highly expressed in liver cancer and non-small cell lung cancer. The OS and disease-free survival of patients with liver cancer and lung cancer with high ITGA7 expression are shorter. This gene promotes the proliferation, invasion, and migration of liver cancer cells and lung cancer cells, and inhibits cell apoptosis [32, 33]. However, no report related to the role of this gene in GC is available.

A study found that ITGA8 is highly expressed in early recurring myeloma and can be used as a potential marker of multiple myeloma recurrence [34]. It is also highly expressed in colon cancer tissues [35]. ITGA8 can also be used to predict the prognosis of clear cell renal cell carcinoma [36]. The result of this study showed that ITGA8 was highly expressed in GC, and its expression was increased with the progression of tumor stages, but it was not associated with the OS of the patients.

The expression of ITGA9 in triple-negative breast cancer is higher than that in other tumors, and the prognosis of patients is significantly worse [37]. ITGA9 is also highly expressed in melanoma tissues and promotes the proliferation and invasion of melanoma cells [38].

Some studies found that tissues with gene variants of ITGA10 and ITGA6 were less susceptible to melanoma [39]. A study analyzed the gene expression profile of 64 cases of primary myxofibrosarcoma and found that patients with high ITGA10 expression had a significantly worse prognosis, revealing that ITGA10 promotes tumor cell survival by activating TRIO/RICTOR signaling. An inhibitor of RAC and mTOR exerts an antitumor effect, revealing a potential treatment strategy for high-risk myxofibrosarcoma patients [40]. Our research results found that ITGA10 had no significant difference in GC.

The bioinformatics analysis revealed that ITGA11 is highly expressed in patients with non-small cell lung cancer and may become a diagnostic and prognostic biomarker [41]. In addition, its high expression in this cancer type is significantly associated to poor prognosis [42]. ITGA11 is also highly expressed in fibroblasts of head and neck cancer [43] and pancreatic cancer [44]. This study found that ITGA11 was highly expressed in GC. The expression of this gene increased with the increase of tumor progression and was related to the poor prognosis of GC patients.

The ITGAD gene is mutated in GC, as revealed by advanced genomic sequencing analysis [45]. The increased variation of ITGAE is associated with the risk of melanoma [39]. The whole exon sequencing of 18 cases of mucoepidermoid carcinoma showed the occurrence of ITGAD gene mutation [46]. The results in this study revealed that the mutation rates of ITGAD, ITGAE, and ITGAL were 5%, 4%, and 5%, respectively. The analysis of ITGAM methylation patterns in GC genome revealed that genome methylation promotes the expression of ITGAM; thus, ITGAM may be a candidate gene for GC diagnosis [13]. ITGAV promotes the proliferation, invasion, and migration of GC cells [14]. ITGAX stimulates angiogenesis in tumors through the overexpression of VEGF2/VEGF-A mediated by PI3K/AKT signaling pathway [47]. Our results showed that ITGAE, ITGAL, ITGAM, and ITGAV were highly expressed in GC, and the expression of ITGAL gene was increased with the increase of GC stages. ITGAV was associated with poor prognosis in patients with GC, and it was the gene with the highest mutation rate of 9% among ITGs.

ITGB1 is upregulated in ovarian cancer. High ITGB1 expression enhances the invasiveness of ovarian cancer cells. In vivo experiments showed that ITGB1 downregulation can inhibit tumor growth and peritoneal metastasis [48, 49]. High ITGB1 expression is also negatively correlated with the prognosis of patients with non-small cell lung cancer [50]. In patients with GC, ITGB1 in the peripheral blood is high, and the later the intermediate stage, the higher the ITGB1 content, which can improve the accuracy of GC diagnosis [51]. ITGB2 is significantly upregulated in serum exosomes in patients with papillary thyroid cancer with lymph node metastasis [52]. ITGB3, ITGB4, and ITGB5 gene polymorphisms are associated with increased GC risk [8]. ITGB4 overexpression was related to the aggressiveness and poor prognosis of many malignant tumors. It is highly expressed in patients with non-small cell lung cancer and may represent a diagnostic and prognostic biomarker [42]. Xu et al. established a biodigital model and found that the measurement of ITGB5 and ITGB1 expression can predict the survival of patients after GC surgery [53]. ITGB6 is significantly increased in the serum of colon cancer, and the level of ITGB6 in the serum of patients after surgery is significantly lower than that before surgery. The higher the level of ITGB6, the lower the survival rate after surgery. The combination of ITGB6 and CEA can be used as a marker for postoperative prognosis monitoring of colon cancer [54]. ITGB6 is highly expressed in oral squamous cell carcinoma and can be used as a diagnostic biomarker [55]. ITGB7 is highly expressed in myeloma cells and tissues, it can regulate the biological processes of multiple myeloma cells such as adhesion, invasion, and migration, and patients with high expression of ITGB7 have a shorter survival time [56]. The bioinformatics analysis revealed that ITGB8 is significantly upregulated in patients with lung adenocarcinoma, which may become a diagnostic biomarker [41]. Further experimental studies showed that ITGB8 silencing inhibits the invasion and migration of lung cancer cells and arrest the cell cycle at G0/*G*1 [57]. ITGB8 is significantly upregulated in ovarian cancer tissues, and the OS and disease-free survival of patients with ovarian cancer with high ITGB8 expression are significantly shortened [58]. Our study found that ITG (B1, B2, B4, B5, B6, and B8) was highly expressed in GC. ITGB2 expression was increased with the progression of GC staging. The OS of GC patients with high ITGB1 expression was significantly shortened compared to the OS in patients with low expression, which was consistent with the basic results of previous studies.

This study has some limitations. First, this study is a single-center study, and the number of samples is small. In the later stage, we need further multicenter, large sample, random, and single-blind study. Second, the poor homogeneity of T3 in gastric cancer samples may be due to the small size of tumor tissues, which were not collected during the sampling process which makes us screen out a relatively small difference in mRNA, but it is sufficient to show that there is indeed a difference in mRNA spectrum between gastric cancer group and adjacent normal tissues group.

In conclusion, this study revealed that the mRNA expression of some ITG genes was closely related to the diagnosis and prognosis of GC. In addition, ITGA5 might be considered as a potential molecular marker in the diagnosis and prognosis of GC. Thus, this work discovered the prognostic value and biological role of ITGA5 in GC. Although these results need further experimental verification, they might help in the understanding of the diagnostic and prognostic function of ITG genes in GC, ultimately contributing to the design of different and potentially effective treatment strategies to combat GC.

## Figures and Tables

**Table 1:** Correlation between the expression of ITG genes and OS in GC patients at different clinical stages.

ITGs (RNA-seq)	Clinical stage: I (*n* = 50); II (*n* = 111); III (*n* = 149); V (*n* = 38)	HR (95% CI)	*P*-value
ITGA1	I	0.85 (0.27–2.68)	0.78
	II	1.14 (0.58–2.25)	0.71
	III	1.51 (0.93–2.45)	0.09
	IV	0.98 (0.42–2.27)	0.96
ITGA2	I	0.45 (0.12–1.7)	0.22
	II	0.86 (0.43–1.7)	0.66
	III	1.12 (0.69–1.8)	0.65
	IV	0.91 (0.39–2.11)	0.83
ITGA3	I	2.65 (0.8–8.81)	0.10
	II	0.59 (0.3–1.18)	0.13
	III	0.82 (0.51–1.32)	0.42
	IV	1.29 (0.57–2.92)	0.55
ITGA4	I	1.15 (0.37–3.57)	0.81
	II	1.04 (0.53–2.05)	0.91
	III	1.04 (0.64–1.68)	0.87
	IV	1.13 (0.49–2.59)	0.77
ITGA5	I	0.71 (0.21–2.42)	0.59
	II	1.37 (0.69–2.7)	0.36
	III	1.66 (1.02–2.71)	0.04
	IV	2.73 (1.15–6.49)	0.02
ITGA6	I	0.69 (0.22–2.19)	0.53
	II	0.46 (0.23–0.94)	0.03
	III	0.79 (0.59–1.27)	0.33
	IV	0.88 (0.38–2.04)	0.76
ITGA7	I	0.83 (0.25–2.73)	0.75
	II	1.15 (0.57–2.29)	0.7
	III	1.01 (0.62–1.62)	0.98
	IV	1.4 (0.6–3.26)	0.43
ITGA8	I	0.75 (0.24–2.39)	0.63
	II	0.85 (0.43–1.68)	0.65
	III	1.53 (0.95–2.48)	0.08
	IV	0.79 (0.34–1.8)	0.57
ITGA9	I	0.96 (0.31–2.98)	0.94
	II	1.4 (0.7–2.77)	0.34
	III	1.42 (0.87–2.29)	0.16
	IV	0.91 (0.39–2.12)	0.83
ITGA10	I	2.1 (0.62–7.2)	0.23
	II	1.48 (0.74–2.97)	0.26
	III	1.43 (0.88–2.23)	0.14
	IV	1.14 (0.49–2.66)	0.76
ITGA11	I	2.04 (0.61–6.78)	0.24
	II	1.45 (0.73–2.87)	0.29
	III	1.42 (0.88–2.31)	0.15
	IV	2.09 (0.88–4.94)	0.09
ITGAD	I	0.52 (0.16–1.7)	0.27
	II	0.83 (0.42–1.65)	0.6
	III	0.76 (0.47–1.24)	0.27
	IV	0.7 (0.29–1.65)	0.41
ITGAE	I	0.72 (0.27–1.93)	0.51
	II	1.07 (0.53–2.14)	0.85
	III	0.95 (0.59–1.54)	0.85
	IV	0.75 (0.33–1.71)	0.49
ITGAL	I	1.34 (0.42–4.25)	0.62
	II	0.84 (0.42–1.67)	0.61
	III	0.94 (0.58–1.51)	0.78
	IV	0.47 (0.19–1.15)	0.09
ITGAM	I	1.6 (0.51–5.04)	0.42
	II	0.67 (0.34–1.33)	0.25
	III	1.17 (0.72–1.9)	0.53
	IV	1.07 (0.46–2.49)	0.87
ITGAV	I	0.69 (0.2–2.32)	0.55
	II	1.74 (0.88–3.46)	0.11
	III	1.95 (1.2–3.17)	<0.01
	IV	3.05 (1.22–7.66)	0.01
ITGAX	I	1.25 (0.4–3.9)	0.69
	II	1.2 (0.6–2.37)	0.61
	III	1.18 (0.73–1.91)	0.51
	IV	1.01 (0.44–2.31)	0.98
ITGB1	I	0.5 (0.14–1.72)	0.26
	II	1.74 (0.87–3.48)	0.12
	III	1.1 (0.68–1.78)	0.69
	IV	2.09 (0.89–4.93)	0.09
ITGB2	I	2.2 (0.66–7.33)	0.19
	II	1.01 (0.51–2.01)	0.97
	III	1.13 (0.7–1.82)	0.62
	IV	0.61 (0.26–1.47)	0.27
ITGB3	I	1.15 (0.37–3.56)	0.81
	II	1.22 (0.62–2.41)	0.57
	III	1.26 (0.78–2.04)	0.35
	IV	1.11 (0.48–2.58)	0.81
ITGB4	I	1.57 (0.5–4.98)	0.44
	II	0.73 (0.37–1.43)	0.36
	III	1.49 (0.91–2.44)	0.11
	IV	0.89 (0.38–2.06)	0.79
ITGB5	I	0.98 (0.31–3.06)	0.97
	II	1.77 (0.88–3.56)	0.11
	III	1.12 (0.69–1.83)	0.63
	IV	2.14 (0.89–5.12)	0.08
ITGB6	I	0.34 (0.09–1.26)	0.09
	II	1.01 (0.52–2)	0.97
	III	1.51 (0.94–2.44)	0.09
	IV	1.15 (0.5–2.66)	0.75
ITGB7	I	0.79 (0.24–2.61)	0.7
	II	1.62 (0.8–3.29)	0.18
	III	1.13 (0.7–1.82)	0.62
	IV	0.71 (0.31–1.63)	0.42
ITGB8	I	0.78 (0.25–2.46)	0.67
	II	0.71 (0.36–1.42)	0.33
	III	0.83 (0.52–1.34)	0.45
	IV	1.65 (0.7–3.86)	0.25

**Table 2:** Correlation between the expression of ITG genes and OS in GC patients at different clinical stages.

ITGs (RNA-seq)	Differentiation: I (*n* = 12); II (*n* = 134); III (*n* = 218);	HR (95% CI)	*P*-value
ITGA1 (none)	Grade I	—	—
	Grade II	1.11 (0.63–1.94)	0.73
	Grade III	1.34 (0.89–2.03)	0.16
ITGA2	Grade I	—	—
	Grade II	0.53 (0.3–0.94)	0.03
	Grade III	1.26 (0.83–1.89)	0.28
ITGA3	Grade I	—	—
	Grade II	0.69 (0.39–1.21)	0.19
	Grade III	1.16 (0.77–1.75)	0.48
ITGA4	Grade I	—	—
	Grade II	1.1 (0.63–1.94)	0.73
	Grade III	1 (0.66–1.5)	0.99
ITGA5	Grade I	—	—
	Grade II	0.97 (0.55–1.69)	0.9
	Grade III	1.43 (0.95–2.16)	0.09
ITGA6	Grade I	—	—
	Grade II	0.52 (0.29–0.93)	0.02
	Grade III	0.8 (0.53–1.21)	0.3
ITGA7	Grade I	—	—
	Grade II	0.93 (0.93–1.63)	0.8
	Grade III	1.08 (0.72–1.63)	0.7
ITGA8	Grade I	—	—
	Grade II	1.13 (0.64–1.98)	0.67
	Grade III	1.07 (0.71–1.61)	0.76
ITGA9	Grade I	—	—
	Grade II	1.1 (0.63–1.93)	0.74
	Grade III	1.19 (079–1.79)	0.4
ITGA10	Grade I	—	—
	Grade II	0.78 (0.44–1.38)	0.39
	Grade III	1.38 (0.91–2.09)	0.13
ITGA11	Grade I	—	—
	Grade II	1.72 (0.96–3.1)	0.07
	Grade III	1.34 (0.88–2.03)	0.17
ITGAD	Grade I	—	—
	Grade II	0.87 (0.49–1.54)	0.63
	Grade III	0.7 (046–1.07)	0.096
ITGAE	Grade I	—	—
	Grade II	2.2 (1.23–3.93)	0.03
	Grade III	0.45 (0.28–0.72)	0.02
ITGAL	Grade I	—	—
	Grade II	1.79 (0.93–3.44)	0.07
	Grade III	0.66 (0.44–1)	0.05
ITGAM	Grade I	—	—
	Grade II	1.54 (0.88–2.7)	0.13
	Grade III	1.39 (0.92–2.12)	0.12
ITGAV	Grade I	—	—
	Grade II	0.51 (0.29–0.92)	0.02
	Grade III	2.34 (1.55–3.54)	<0.01
ITGAX	Grade I	—	—
	Grade II	1.7 (0.97–3.01)	0.06
	Grade III	0.99 (0.65–1.49)	0.95
ITGB1	Grade I	—	—
	Grade II	0.65 (0.37–1.15)	0.13
	Grade III	1.59 (1.05–2.42)	0.02
ITGB2	Grade I	—	—
	Grade II	1.2 (0.68–2.1)	0.53
	Grade III	0.85 (0.56–1.28)	0.43
ITGB3	Grade I	—	—
	Grade II	1.01 (0.58–1.77)	0.97
	Grade III	1.49 (0.98–2.26)	0.06
ITGB4	Grade I	—	—
	Grade II	0.83 (0.47–1.46)	0.5
	Grade III	1.09 (0.72–1.64)	0.7
ITGB5	Grade I	—	—
	Grade II	1.12 (0.64–1.97)	0.69
	Grade III	1.54 (1.01–2.35)	0.05
ITGB6	Grade I	—	—
	Grade II	0.9 (0.51–1.6)	0.73
	Grade III	1.22 (0.81–1.83)	0.35
ITGB7	Grade I	—	—
	Grade II	1.28 (0.73–2.26)	0.39
	Grade III	0.94 (0.64–1.45)	0.85
ITGB8	Grade I	—	—
	Grade II	0.92 (0.52–1.62)	0.77
	Grade III	1.01 (0.67–1.53)	0.95

## Data Availability

The data used to support the findings of this study are included within the article.
